# Society Meetings

**Published:** 1897-02

**Authors:** 


### Society Meetings.

The Lake Erie Medical Society held its regular meeting at North Collins, Thursday, January 14, 1897. The following papers were presented: (1) Mania a potu, by Dr. S. S. Bedient, Little Valley; discussed by Drs. Beals, Deck and Putnam. (2) Whooping cough, by Dr. J. Cherry, Eden; discussed by Drs. Davis, Rugg and Lattin. (3) Notes on post-graduate work, by Dr. B. S. Bourne, Hamburg; discussed by Drs. Ward, Holden and Coss. (4) Treatment of Pott's disease, by Dr. W. J. French, Hamlet; discussed by Drs. Teft, Thompson and Robbins.

The officers are: Dr. A. D. Lake, president, Gowanda; Dr. N. E. Beardsley, vice-president, Dunkirk; Dr. W. W. Jones, secretary and treasurer, Dayton. Program committee, Dr. W. M. Ward, North Collins, and Dr. T. T. Clohessy, North Evans.

---

The Medical Society of the County of Chautauqua held its regular semi-annual meeting at Fredonia, Tuesday, January 12, 1897. There was a large attendance and the program was carried out with one exception—Dr. Parmenter, of Buffalo, telegraphed his inability to be present. A profitable discussion was held upon the papers, and a large number of clinical cases were reported. The next meeting of the society will be held on the second Tuesday of July, at Chautauqua, at which time a banquet will be given in the evening.

---

The Buffalo Academy of Medicine held meetings during the month of January as follows:

*Section on Medicine.*—Tuesday evening, January 5th, program: Comparative prevalence of insanity, by Dr. H. G. Hopkins; Heredity as a cause, by Dr. William C. Krauss; Newer pathology, by Dr. H. G. Matzinger; Symptoms of incipient insanity, by Dr. James W. Putnam; State care of the insane, by Dr. Floyd S. Crego; Prognosis of insanity, by Dr. Arthur W. Hurd; Newer methods of treatment, by Dr. Harry A. Wood. A case of syringomyelia was presented by Dr. Crego.

*Section on Surgery.*—Tuesday evening, January 12th, program: Surgical treatment of perforating typhoid ulcer, by T. M. T. Finney, associate professor of surgery, Johns Hopkins Hospital, Baltimore; Discussion from a medical standpoint, by Dr. Charles Cary; Discussion from a surgical aspect, by Dr. M. Hartwig; The history of bleeding, by Dr. James W. Putnam; Presentation of cases by Dr. Herman Mynter.

*Section on Pathology.*—Tuesday evening, January 19th, program: Exhibition of specimens; Large aneurism of the aorta, by Dr. De Lancey Rochester; Other specimens by Dr. E. P. Lothrop and Dr. H. E. Hayd.

[The Journal went to press before the meeting of January 25th.]

---

The Tri-State Medical Society of Iowa, Illinois and Missouri will hold its fifth annual meeting at St. Louis, April 6, 7 and 8, 1897. A large number of valuable papers will be read. Dr. Joseph Price, of Philadelphia, will hold the surgical clinic, Dr. James T. Whittaker, of Cincinnati, the medical clinic, and Dr. Dudley Reynolds, ophthalmic clinic. Dr. G. Frank Lydston, of Chicago, will entertain the members with an original story during one of the evening sessions. The officers are: Dr. A. H. Cordier, president, Rialto Bldg., Kansas City; Dr. Hugh T. Patrick, 1st vice-president, Chicago; Dr. H. C. Eschbach, 2d vice-president, Albia, Ia.; Dr. G. W. Cale, secretary, 4403 Washington boulevard, St. Louis;

Dr. C. S. Chase, treasurer, Waterloo, Ia. The preliminary program will be issued soon.

---

The Medical Society of the County of Westchester held its one hundredth annual meeting at Yonkers, on Wednesday, January 20, 1897. The gathering partook of the nature of a centennial anniversary. There was a large attendance of physicians and many valuable papers were read. The meeting was held under the presidency of Dr. Archibald M. Campbell, of Mt. Vernon. Dr. William M. Carhart, of Peekskill, read a paper on The importance of early correction of refractive errors of school children ; Dr. Charles McBurney, of New York, read a paper on Appendicitis. After dinner had been served, Mayor Peene made an address, and then Dr. Daniel Lewis, of New York, president of the state board of health, addressed the society under the title Stamping out tuberculosis : how to solve the problem. Dr. A. T. Banning, the coroner of the county, and Dr. Edward F. Brush, both of Mt. Vernon, took part in the discussion.

This very successful anniversary meeting was arranged under the supervision of Dr. E. M. Morrell, of Yonkers, chairman of the committee of arrangements, who received a vote of thanks for his successful labors.

The Medical Society of the County of Westchester was organised in 1797, and is, therefore, the oldest county society of the state.

